# Correction: “Betaone” barley water extract suppresses ovariectomy-induced osteoporosis *in vivo* and RANKL-induced osteoclast differentiation *in vitro*

**DOI:** 10.1371/journal.pone.0339919

**Published:** 2025-12-30

**Authors:** Yongjin Lee, Hyun-Jin Lee, Kwang-Jin Kim, Han-Byeol Shin, Yoon-A Shin, Holim Jin, Ju Ri Ham, Soo-Young Choi, Mi-Ja Lee, Mi-Kyung Lee, Young-Jin Son

In Fig 3, the Estrogen (pg/mL) graph should not be included. Please see the correct Fig 3 here.

**Fig 3:**
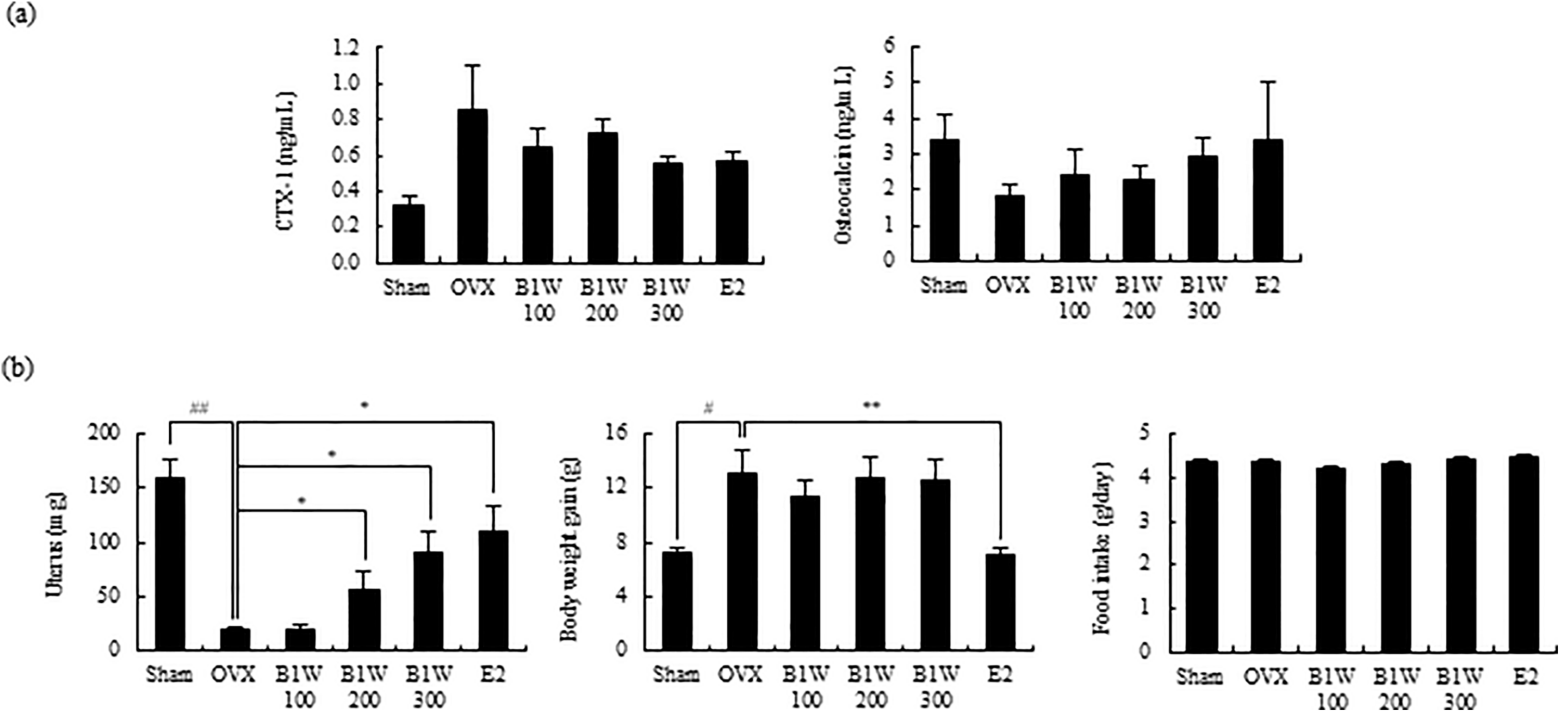
The effect of B1W on (a) serum biomarkers, and (b) weight of uterus, body weight gain and food intake in OVX mice. Data are presented as the mean ± SEM. #P < 0.05, ##P < 0.01, versus the Sham group; *P < 0.05, **P < 0.01, ***P < 0.001, versus the OVX group.
