# Supplementary figures and images for: “Betaone” barley water extract suppresses ovariectomy-induced osteoporosis in vivo and RANKL-induced osteoclast differentiation in vitro
Source: PLoS One. 2025 Feb 21;20(2):e0317894. doi: 10.1371/journal.pone.0317894 (PMC11844866; doi:10.1371/journal.pone.0317894)

nFATc1

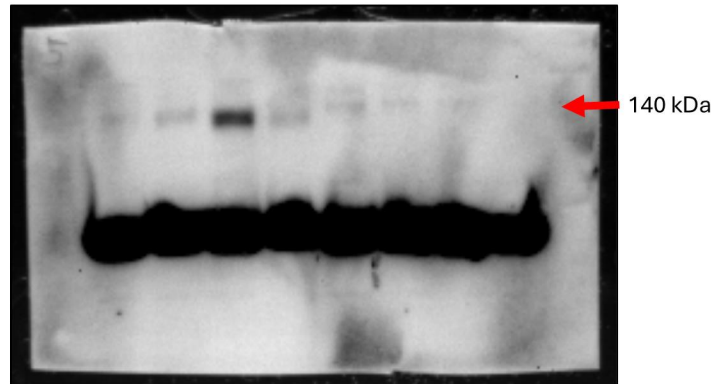

$\beta$ -actin

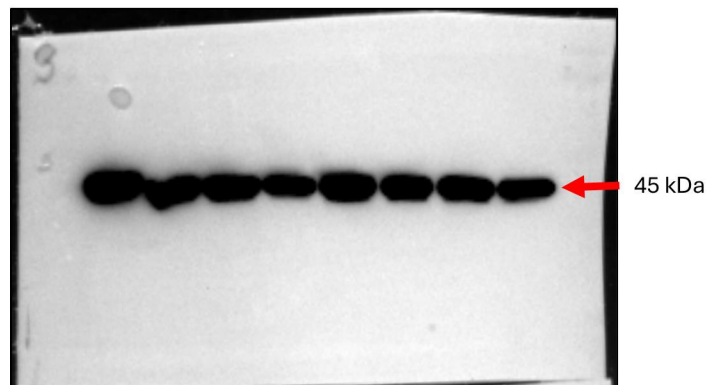

p-JNK

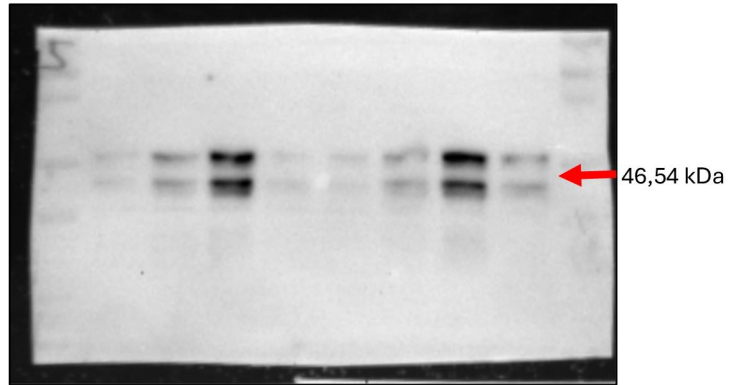

t-JNK

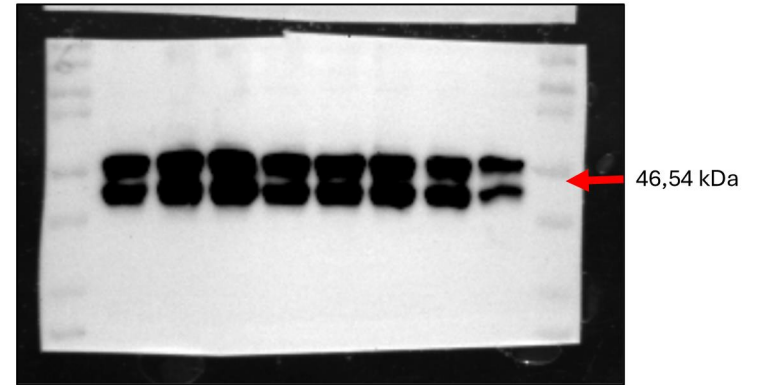

p-p38

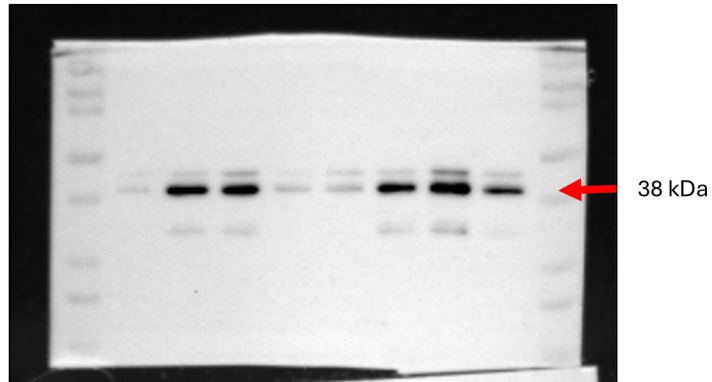

t-p38

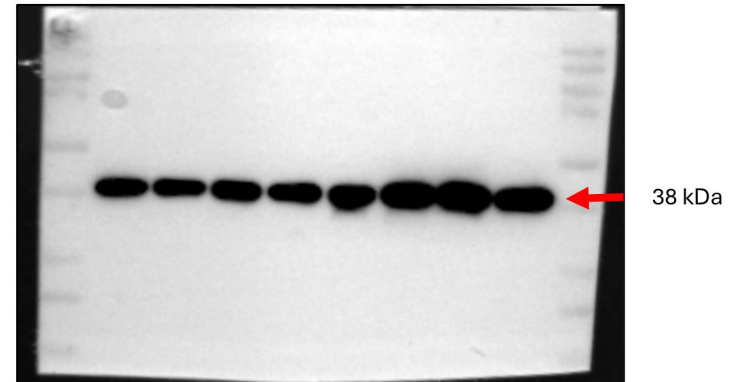

$\beta$ -actin

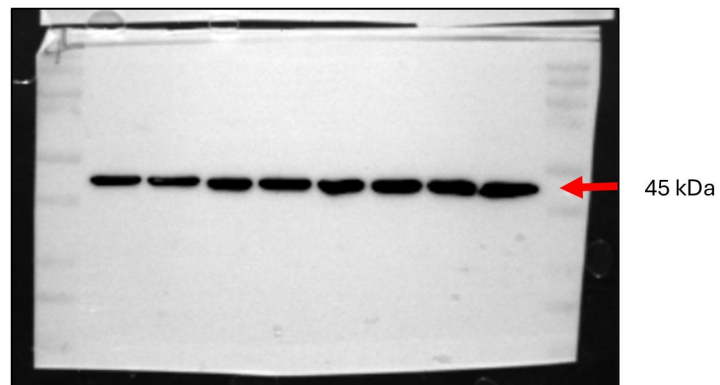

Supplement: S1 Fig — (PDF) [file pone.0317894.s001.pdf]
