# Supplementary material for: “Betaone” barley water extract suppresses ovariectomy-induced osteoporosis in vivo and RANKL-induced osteoclast differentiation in vitro
Source: PLoS One. 2025 Feb 21;20(2):e0317894. doi: 10.1371/journal.pone.0317894 (PMC11844866; doi:10.1371/journal.pone.0317894)
Supplement: Attachment — Submitted filename: PLOS ONE-Response for Comments.docx [file pone.0317894.s002.docx]

*Editor-in-Chief*	Dec 13, 2024

*PLOS ONE*

Dear Editor:

Please find enclosed our manuscript entitled ““Betaone” barley water extract suppresses ovariectomy-induced osteoporosis in vivo and RANKL-induced osteoclast differentiation in vitro” (PONE-D-24-41839) which we request you to consider for publication as an *Original Article* in *PLOS ONE.*

**Reviewer #1 : Comments and Suggestions for Authors**

93-96: The yield of B1W was approximately 10.8%. Please provide the extraction yield report for B1W.

**Ans.) We added the extraction yield in the Materials and Methods. (400g of B1 powder was extracted with 3.6 L of distilled water and concentrated, and approximately 43.202 g of extracted powder was obtained. The yield of the B1W was about 10.8%., Line 95-97)**

99-112: Please include ultra-performance liquid chromatography data for TH and FA, not just graphs.

**Ans.) I added UPLC chromatogram(Figure 1) and results(Line 207-211) as you advised.**

117-120: Were the rats 5 weeks old at the time of modeling? Female rats at this age are not yet sexually mature, which may not be the optimal time for ovariectomy.

**Ans.) We purchased 5-week-old female ICRs and acclimated them for 1 week before performing the OVX surgery. The 6-week-old mice have well-developed ovaries, and we considered that there is no problems with the experiments. According to the mouse and human age comparisons (Dutta S, Sengupta P. Men and mice: Relating their ages.  *Life Sci* . 2016;152:244-248. doi:10.1016/j.lfs.2015.10.025):**

**A 1-week-old mouse is roughly equivalent to a 1-year-old human.**

**A 6-week-old mouse corresponds to an 18- to 20-year-old human and is considered sexually mature.**

**A 1-year-old mouse is approximately equivalent to a 30- to 40-year-old human.**

**A 2-year-old mouse is comparable to a 70- to 80-year-old human and is considered elderly.**

**Also, other researchers performed the experiment with 6-week-old OVX model mice below references.**

**[1) Govoni KE, Wergedal JE, Chadwick RB, Srivastava AK, Mohan S. Prepubertal OVX increases IGF-I expression and bone accretion in C57BL/6J mice. *Am J Physiol Endocrinol Metab*. 2008;295(5):E1172-E1180. doi:10.1152/ajpendo.90507.2008.**

**2) Kitajima Y, Ogawa S, Egusa S, Ono Y. Soymilk Improves Muscle Weakness in Young Ovariectomized Female Mice. *Nutrients*. 2017;9(8):834. Published 2017 Aug 4. doi:10.3390/nu9080834]**

125-135: The experimental animals were sacrificed 6 weeks post-surgery, and serum was extracted for bioanalysis. What method was used to collect the blood? Is it venous blood? - heart extraction

**Ans.) We obtained the sufficient blood samples for physiological analysis using a heart extraction, and we added this procedure to the Materials and Methods section(Line 127-129).**

139-147: BMMSCs were extracted from 5-week-old male rats, which may be somewhat older, potentially affecting the activity of rat bone marrow cells. How were BMMs pretreated with B1W? What passage number were the subsequent BMMSCs used for cell experiments?

**Ans.) B1W was treated at the next day after BMMSCs seeding. We performed in vitro cell study using the enough quantities of cells (40×10^6^). The passage number of BMMSCs used was 1.**

157-160: In the cytotoxicity experiments, was 10 ng/ml RANKL used to induce osteoclasts for four days?

**Ans.) We performed the cytotoxicity experiments without RANKL treatment. We identified the cytotoxic effect during the proliferation of BMMs treated M-CSF. In the osteoclastogenesis research, the cytotoxicity experiments are usually used using BMMs without RANKL treatment like below references.**

**[1]Wu X, Huang L, Liu J. Effects of adiponectin on osteoclastogenesis from mouse bone marrow-derived monocytes. Exp Ther Med. 2019;17(2):1228-1233. doi:10.3892/etm.2018.7069,**

**2) Qu Y, Liu X, Zong S, Sun H, Liu S, Zhao Y. Protocatechualdehyde Inhibits the Osteoclast Differentiation of RAW264.7 and BMM Cells by Regulating NF- κ B and MAPK Activity [retracted in: Biomed Res Int. 2023 Nov 29;2023:9820815. doi: 10.1155/2023/9820815]. Biomed Res Int . 2021;2021:6108999. Published 2021 Jul 16. doi:10.1155/2021/6108999,**

**3) Chen S, Chu B, Chen Y, et al. Neferine suppresses osteoclast differentiation through suppressing NF-κB signal pathway but not MAPKs and promote osteogenesis. J Cell Physiol . 2019;234(12):22960-22971. doi:10.1002/jcp.28857,**

**4) Zhang S, Huo S, Li H, et al. Flufenamic acid inhibits osteoclast formation and bone resorption and act against estrogen-dependent bone loss in mice. Int Immunopharmacol . 2020;78:106014. doi:10.1016/j.intimp.2019.106014]**

182：In Table 1, what does "sense" and "anti-sense" refer to? The formatting appears incorrect.

**Ans.) As you advised, I corrected the terms, forward and reverse instead of sense and anti-sense.**

207: Table 2 is not formatted correctly.

**Ans.) We modified as advised.**

273-277: The timing and dosage of RANKL induction were not annotated, nor are they indicated in the corresponding Fig. 5.

**Ans.) We described the timing and dosage of RANKL to differentiate osteoclast in Materials and Methods (Line 187-189).**

What is the difference between bone pit formation and TRAP staining? The results of tissue section staining for osteoporosis should be supplemented for verification.

**Ans.) Bone pit assays evaluate the functional activity of osteoclasts by measuring their bone resorption capacity. Meanwhile, we can identify the osteoclasts and visualize TRAP-positive multinucleated cells. So, we think the confirmation of the resorption activity of bone pit assay and the identification of TRAP stained osteoclasts is enough to provide the activity of B1W against osteoclastogenesis. We are going to plan to perform the tissue section staining for osteoporosis for the double-check verification.**

Fig. 3: TRAP-stained positive cells should appear red, but the coloration in the article appears biased. Additionally, no scale bars are marked in panels A and D.

**Ans.) The color of TRAP-stained positive cells is seen in red or similar to violet. The color of our result was similar to violet. According to other reference, the color is similar to ours like below.**

**And the scale bars was inserted in the figure 4 panels (a) and (d).**

**(Gu JH, Tong XS, Chen GH, et al. Regulation of matrix metalloproteinase-9 protein expression by 1α, 25-(OH)₂D₃ during osteoclast differentiation. J Vet Sci. 2014;15(1):133-140. doi:10.4142/jvs.2014.15.1.133)**

**(Chevalier C, Çolakoğlu M, Brun J, et al. Primary mouse osteoblast and osteoclast culturing and analysis. *STAR Protoc*. 2021;2(2):100452. Published 2021 Apr 13. doi:10.1016/j.xpro.2021.100452)**

**Reviewer #2: Comments and Suggestions for Authors**

123：The referenced study primarily investigates the effects of 17α estradiol, prompting consideration for replacing literature that examines 17β estradiol. This substitution would enhance the experimental rationale surrounding the use of 17β estradiol.

**Ans.) I added references (reference number 20, 21) as you advised.**

232、233：It is possible that the designation of "Fig. 2a" instead of "Fig. 1a" in reference to the picture could be a clerical error; please take note of this correction.

**Ans.) I corrected it as comment (Line 238-239).**

274：Based on the experimental results from WB, the statement that the expression level of NFATc1 in the control group increases over time is not entirely accurate.

**Ans.) I corrected the mistyping in the sentence (Line 280-282).**

**Reviewer #3: Comments and Suggestions for Authors**

The study by Yongjin Lee et al. explored effects of B1W on ovariectomy (OVX)-induced bone loss in vivo and osteoclastogenesis in vitro. The paper is well-written and presents promising results. However, there are a couple of concerns to make this study more convincing: Please add associated biomechanical testing data to double confirm B1W-induced anti-osteoporosis. Please supplement relevant data on effects of B1W on osteogenesis and bone formation.

**Ans.) We performed the experiment for osteoblast activity of B1W. As the below figure, it was confirmed that there was no osteoblast activity of B1W. So, we could not proceed the further study for osteoblast activity.**

**Fig) Osteoblast Activity of B1W. There is no activity at high concentration, 100 μg/mL of B1W.**
